# Dysfunctional problem-based learning curricula: resolving the problem

**DOI:** 10.1186/1472-6920-12-89

**Published:** 2012-09-25

**Authors:** William K Lim

**Affiliations:** 1 Department of Paraclinical Sciences, Faculty of Medicine and Health Sciences, Universiti Malaysia Sarawak, Lot 77, KTLD Section 22, Jalan Tun Zaidi Adruce, Kuching, Sarawak, 93150, Malaysia

## Abstract

**Background:**

Problem-based learning (PBL) has become the most significant innovation in medical education of the past 40 years. In contrast to exam-centered, lecture-based conventional curricula, PBL is a comprehensive curricular strategy that fosters student-centred learning and the skills desired in physicians. The rapid spread of PBL has produced many variants. One of the most common is 'hybrid PBL' where conventional teaching methods are implemented alongside PBL. This paper contends that the mixing of these two opposing educational philosophies can undermine PBL and nullify its positive benefits. Schools using hybrid PBL and lacking medical education expertise may end up with a dysfunctional curriculum worse off than the traditional approach.

**Discussion:**

For hybrid PBL schools with a dysfunctional curriculum, standard PBL is a cost-feasible option that confers the benefits of the PBL approach. This paper describes the signs of a dysfunctional PBL curriculum to aid hybrid PBL schools in recognising curricular breakdown. Next it discusses alternative curricular strategies and costs associated with PBL. It then details the four critical factors for successful conversion to standard PBL: dealing with staff resistance, understanding the role of lectures, adequate time for preparation and support from the administrative leadership.

**Summary:**

Hybrid PBL curricula without oversight by staff with medical education expertise can degenerate into dysfunctional curricula inferior even to the traditional approach from which PBL emerged. Such schools should inspect their curriculum periodically for signs of dysfunction to enable timely corrective action. A decision to convert fully to standard PBL is cost feasible but will require time, expertise and commitment which is only sustainable with supportive leadership.

## Background

Problem-based learning (PBL) is the single most important innovation in medical education of the past 40 years [1]. It was developed at McMaster university in response to teacher-centered and discipline-based preclinical medical education prevalent in the 1960s, where students receive teacher-determined material by lectures for reproduction in factual tests [2]. This short term cramming of large amounts of information organized around isolated subjects did not favor recall in the clinical years. It was neither preparing students to solve clinical problems nor to become self-directed lifelong learners. In contrast, PBL students in tutor-guided small groups attempt to resolve a real-life clinical problem by using their existing knowledge to generate hypotheses and then actively finding the cross-disciplinary knowledge they need to fully understand the problem [3]. Hence PBL is a constructivist [4], student-centered and problem-based approach to medical education. It is geared to facilitate knowledge retention and application while fostering the skills desired in physicians, such as clinical reasoning, critical thinking and self directed learning [2]. The PBL approach has been found to improve physician competency in the social and cognitive domains [5].

As the McMaster model became known, staff from Maastricht (Netherlands) and Newcastle (Australia) spent time at McMaster in the 1970s before returning to implement PBL at their new medical schools [6]. In 1979, The University of New Mexico medical school offered a PBL curriculum as an alternative track [7]. Over the next two decades, established schools like Harvard [8], Sherbrooke [9] (Canada), Manchester and Liverpool (U.K.) [10] changed their traditional curricula to incorporate PBL. In 2003, 70% of U.S. medical schools used PBL in the preclinical years to some extent [11]. As medical schools worldwide adapted PBL into their curricula, variants arose depending on the school they modeled upon, staff preference and local constraints. This resulted in a diversity of PBL models described as ranging from full [12] to near-full [13] , partial [14] or hybrid [15]. Taylor and Miflin concluded that after 40 years of dissemination and evolution, PBL was a genus with many species, many of which “have been found wanting in terms of the initial promise” [16].

Among the leading debates on what constitutes PBL has been the implementation approach (method or philosophy) and the basic type (pure or hybrid).

### 1. Method of philosophy

While some schools revamped their curriculum to incorporate the PBL approach, others make that claim by simply adding PBL tutorials onto an otherwise unchanged conventional curriculum. However, PBL’s pioneers understood it as “a whole curriculum, not a teaching method that can be used alongside other methods” [2]. In setting the ground rules for true PBL, Maudsley emphasized that it is both “method and philosophy”, a comprehensive curricular strategy to be supported, and not undermined, by other curricular elements [1].

### 2. Pure or hybrid

The term ‘hybrid’ PBL is thought to originate from Harvard’s New Pathway curriculum in which “the scope, frequency and format of lectures and laboratory sessions could be effectively altered to dovetail with active problem-based discussions” [8]. A 2003 survey of US medical schools showed that for PBL schools, almost one-half allocated a mere 10% or less of staff-student contact hours for PBL [11], showing that PBL was mostly hybridized with other curricular inputs. In Walton and Matthews’ summary of PBL essentials, “Lectures…Seminars, laboratory demonstrations and laboratory exercises are essential, and can be integrated into any PBL curriculum..each selected and timed to help towards the attainment of specific objectives” [17]. Albanese and Mitchell’s review of PBL implementation gave one of the conditions that facilitate PBL as “Small-group tutorials and independent study constitute the main instructional activities. Other instructional methods (lectures, labs, clinical skills sessions) are not eliminated but are kept to the minimum and are coordinated with the patient problems” [18]. In the most comprehensive review yet on the issue of ‘hybrid PBL’, Kwan and Tam suggested that a ‘pure’ form of PBL is now practically non-existent [19]. Therefore, I propose the term ‘standard PBL’ to describe PBL curricula where lectures and other didactic sessions are judiciously used to support the active, self directed and student-centered learning triggered by problem scenarios. ‘Hybrid’ PBL would then refer to all curricula incorporating PBL-style tutorials but not fitting the criteria for standard PBL.

Kwan and Tam [19] divided hybrid PBL into 4 subtypes: Type I is a conventional curriculum incorporating 2–3 PBL problems per academic year, a change which the authors characterized as merely cosmetic. This is contrasted with type IV where PBL is “the main learning platform” supplemented by unconventional (student-centered and interactive) lectures to enrich the students’ self directed learning. I have termed this as standard PBL, and they placed the McMaster model in this category. Types II and III are lecture-based curricula where Type II incorporates PBL tutorials for supplementary knowledge while type III uses PBL problems for applying lecture-delivered information. The authors described these as PBL curricula that have deviated from the PBL philosophy. I propose the term ‘dysfunctional PBL’ to characterise curricular weakness due to faulty PBL implementation.

## Discussion

In a standard PBL curriculum, lectures and other inputs serve as instructional scaffolding to support learning by the PBL approach. In contrast, lectures in a conventional curriculum are intended for content experts to directly transmit knowledge to the students. While it is common to add more lectures to a lecture-based curriculum, excessive didactic sessions in a PBL curriculum conflicts with its student-centered, independent learning philosophy. Despite the prevalence of hybrid PBL, there is scant literature on how to diagnose and manage a dysfunctioning hybrid PBL curriculum. In this article, I describe the signs of a dysfunctional PBL curriculum to aid hybrid PBL schools assess the state of their curriculum. This is followed by a review of the issues surrounding adoption of PBL in the light of resource requirements and alternative curricular strategies. I then discuss the critical issues and steps for steering a failing curriculum back towards standard PBL.

## Signs of dysfunctional PBL curricula

Schools with hybrid PBL curricula that lack curriculum development expertise should undertake regular inspection of their curricula for one or more of the following signs of dysfunction:

### 1. Presence of curriculum components that undermine the PBL philosophy

PBL sessions may exist alongside lectures such that over the course of a preclinical year, students tackle weekly multidisciplinary problems and yet attend hundreds of discipline-based lectures which often cover the same ground. If the week’s lecture contents effectively ‘solve’ the trigger problem, the active inquiry processes fostered by PBL will be subverted [20]. This content overlap can also reduce the second and subsequent PBL sessions into a mere regurgitation of lecture notes. When the day is packed with lectures, students lack time and motivation for meaningful self directed learning. It is noteworthy that the Harvard New Pathway curriculum leaves three afternoons every week free of scheduled classes to enable preparation for the PBL sessions [10]. There are also schools that conduct a whole-class, post-PBL lecture for the case writer to fill up any knowledge gaps among the students. This discourages independent study and opposes PBL’s active learning approach.

Where summative assessments are frequent and fact-based, as they usually are in lecture-based curricula, students are driven to eschew deep learning approaches and favor cramming to maximize their grades, a scenario which PBL was designed to avert. Where the lecture load is heavy and staff are required to contribute assessment questions from the lectures they delivered, the study of lecture contents alone is sufficient to pass the assessments. Since the contribution of PBL to final grades may just be a small percentage awarded for attendance at PBL tutorials, students will not be motivated to put effort into PBL aside from attending the sessions. Such a hybrid curriculum is in essence a conventional lecture-based curriculum except that students have the extra burden of going through the motions of attending PBL sessions.

### 2. The PBL component is carried on without maintenance

Schools with a dysfunctional PBL curriculum often lack medical education expertise. Staff are usually unaware of PBL’s curricular philosophy and typically view PBL as one of many teaching methods. Hence little or no resources is allocated for its maintenance or monitoring. In such schools, PBL problems tend to be reused without review, thus perpetuating any errors or content overlap with other didactic sessions. If student numbers increase without recruitment of more tutors, the size of PBL groups would inevitably increase beyond the typical recommended maximum of 8 to 10 students [21]. This hinders student participation and the tutor’s ability to monitor individual group members, thus weakening the group process. Where PBL is regarded as an optional add-on to lectures, PBL sessions may be reduced or dropped due to shortfall in rooms or tutors, and when the class schedule becomes too packed.

### 3. Curriculum reviews occur without reference to the PBL component

The implementation of PBL is often opposed by staff favoring the conventional curriculum. Curriculum review meetings are often used by PBL opponents to add more discipline-specific teaching sessions. They may request new lectures by citing disappointing assessment results from topics learnt through PBL. However, poor results from lecture-taught topics never lead to the removal of those lectures or their conversion into PBL problems. Such undermining of PBL may go unchallenged at preclinical curriculum reviews when basic science staff are invited to attend as a representative of their discipline, with no one assigned to represent PBL.

### 4. Faculty development programs do not teach PBL as an overall curriculum strategy

Schools that do not accept PBL as an overall curriculum strategy often perpetuate the situation through their staff development programmes. Many tutor training workshops merely teach the tutorial process without presenting PBL as a whole-curriculum philosophy. Tutors trained in this manner may regard PBL groups as no different from the small groups they tutored in conventional curricula. Hence these tutors may operate by frequently intervening with their own didactic questions and then supplying the answers, thus disrupting the PBL process. Students will also be unmotivated to put effort in analyzing a problem if the tutors see themselves as information providers whose job it is to disseminate the problem’s learning objectives.

Where case-writing workshops do not emphasize the difference between PBL and other teaching methods, case writers may perceive PBL problems as performing the same role as standalone clinical cases used during clerkships. Hence they may write scenarios which are not open-ended problems that stimulate inquiry, but “a simple problem with a well-defined solution, which results in a scavenger hunt for information from resources that the professor has provided” [22]. Ignorance of PBL as a whole curriculum strategy may lead writers to construct problems as independent entities, disregarding content overlap with the week’s scheduled classes or duplication of the preceding problems’ learning objectives. In such curricula, PBL is reduced to adjunct teaching that may keep repeating certain curriculum content while leaving other content out completely.

Kwan and Tam cited a quote that “Poor teaching is bad, but poor PBL is even worse” [19]. In a lecture-based curriculum where the lectures are poor, the students can still study the lecture content themselves, but if PBL tutorials are poor, the students will be demotivated and little new knowledge will be constructed. Implementing PBL on top of a traditional lecture-based program is akin to telling students that they must actively construct knowledge, but subsequently delivering it to them via comprehensive lectures. The implication on the faculty is either they do not understand pedagogical principles or they view their students as incapable of self directed learning [23]. In such a mixed and congested curriculum, many students end up detesting PBL.

## Alternative curricular strategies and cost-controlled PBL implementation

Schools with a dysfunctional PBL curriculum have the option of returning to the traditional curriculum, adopting standard PBL or some other curricular model. PBL is often regarded as being resource intensive. While cost is a constraint, an ineffective curriculum is equally unacceptable. In contrast to the traditional curriculum, PBL's curricular approach meets the recommendations of accrediting bodies such as the Liaison Committee on Medical Education (independent study and active learning) [24] and the General Medical Council (UK) (self directed, small-group oriented learning with integration of basic and clinical sciences) [25]. In recent times, case-based learning (CBL) and team-based learning (TBL) have been promoted as cost-effective models for small group learning. Unlike PBL, both CBL and TBL are not whole curriculum philosophies but approaches that can be incorporated in a whole or a part of a course. They introduce elements of active learning, problem solving and group work within a teacher-directed framework. Replacement of PBL with either CBL or TBL will require extensive faculty training and a large time outlay for designing case modules.

### 1. Comparing PBL with CBL and TBL

In CBL, students in small groups use knowledge from advance reading to solve a clinical problem under the direction of a facilitator familiar with the subject matter [26]. CBL can be incorporated into a PBL curriculum, either as occasional case-based exercises [27] or a separate longitudinal course [28] aimed at applying knowledge to solve authentic problems. TBL has been advocated as a supplement to, and replacement for PBL [29]. In TBL, small groups in a large class setting do prior reading to take tests and solve problems collaboratively with input from a content expert facilitator. Unlike PBL or CBL, only a single facilitator is required for the whole class. TBL has been used in a PBL school for improving clinical reasoning ability prior to clinical clerkships [30] and to prepare premed students for PBL in medical school [31]. Sets of lectures in a traditional curriculum can be converted into a TBL format for more active learning. Both CBL and TBL incorporates didactic teaching so that replacement of PBL with either approach represents a shift along the continuum towards teacher-centered learning. Of the three, PBL's multidisciplinary open inquiry approach is the most student centered and well placed to nurture self directed and lifelong learning.

### 2. Cost feasible PBL implementation

Although PBL is described as a resource intensive curriculum model, there are few studies that compare costing for different models [32]. Most of the start-up cost is for construction of tutorial rooms, which is already available in schools that started with PBL. This cost can be minimized by using each room for several groups and multiple purposes. This strategy allowed a school with 240 students spread over two preclinical years, with each tutorial group meeting thrice weekly, to manage with only 8 PBL rooms [33]. In this school, PBL facilities accounted for under 5% of total infrastructure costs. Room requirements can also be minimized by not going below the norm of 8–10 students per group. As a trade-off for tutorial rooms, PBL schools may not need as many large sized lecture theatres as traditional schools. A study in a school undergoing curricular change found that scheduled teaching time of the faculty would not rise upon conversion from a lecture-based to a PBL-based curriculum [34]. Tutor salaries was reported to account for almost 90% of recurring financial costs of a PBL school that employed part time tutors, an outlay that does not apply to schools utilizing its own faculty as tutors [33]. The use of senior students as tutors deserve further exploration [35]. PBL schools can use other cost-effective measures like an e-learning platform to deliver trigger materials and podcasts for lectures. However, PBL is preferably started in Year 1 so that students can adapt quickly to student centered learning. Thus PBL is a whole curriculum strategy that meets the current criteria for effective medical education. Though it could incur more resources, there are ways to maintain costs at a minimum.

## Resolving the Problem

A dysfunctioning hybrid PBL curriculum is inferior even to the conventional curriculum from which PBL arose. Schools that want to resolve this problem and derive the benefits of PBL should convert to standard PBL. Successful conversion will depend on the able handling of 4 critical factors: the nature of resistance to PBL, use of lectures in standard PBL, importance of adequate preparation and the necessity for supportive leadership.

### 1. Resistance

A large part of resistance towards PBL is natural resistance against change, as would be expected from staff who have spent an entire career in lecture-based curricula. For them, changing to PBL would mean giving up years of accumulated lecture materials to become a novice small group tutor. Also, many think little of PBL because they had never understood it as a comprehensive curriculum philosophy, but as just one of many methods to run small group tutorials. Still others have been in schools where aggressive PBL proponents effected a curriculum change to PBL without spending sufficient time selling it to them. Consequently, the mere mention of PBL can evoke responses ranging from polite disagreement to vehement loathing.

At a more fundamental level, Margetson has expounded on hostility to PBL arising from one’s conception of how knowledge is acquired [36]. Some PBL opponents presume that the only way to gain knowledge is through direct transmission of information by a content expert. This notion conflicts with PBL’s premise that educational discoveries can be made if facilitated by appropriate structures of inquiry and critical reflection. Given the chance, holders of the instructivist view would reject PBL or else introduce more lectures into a PBL curriculum. If most staff are of this mindset, those in favor of converting to standard PBL would need to prepare for a long and hard campaign. The conversion at Harvard was accomplished after a “long prolegomenon to win hearts and minds. Only then did the battle commence” [37]. A Norwegian school reported that failure to prepare for resistance from staff could result in a compromised hybrid model far short of standard PBL [38].

### 2. Lectures

Hamdy has succinctly stated that “Having lectures or resource sessions given by faculty to support students’ learning is not against the PBL religion” [39]. Barrows viewed lectures as an efficient way for an expert to distil difficult subjects into easily digested capsules for large group delivery, especially if the lecturer is also a good communicator [2]. Taylor and Miflin recommended that PBL problem writers design the supporting lectures, explain the rationale to the lecturers and monitor the delivery of the lectures [16]. Unlike content-covering lectures in lecture-based curricula, those in a PBL curriculum play specific support roles [40]. Introductory lectures can be given at the start of every new teaching module to give an overview of the basic concepts, technical jargon and helpful learning resources. It can also include the boundaries of learning in the module to guide students from veering beyond the scope of the curriculum. The delivery of such lectures before the problem scenario does not oppose Barrows’ principle that “The problem is encountered *first* in the learning process” [2] because it serves to scaffold the students’ ability to fully engage the problem when they receive it. Summary lectures at the conclusion of the module can show how the knowledge learnt is important in clinical practice, how it relates to other modules [41] and highlight recent significant research impacting the field. Other lectures can address important issues outside the scope of the problem scenarios, or elaborate on complex topics [42]- a task particularly suited for faculty that can inspire love for the field and make complex concepts easily understandable [17].

Lectures in a PBL curriculum should be consistent with the PBL philosophy of active, deep and self directed learning. Lectures can foster self directed learning when students know the contents will help them to fully engage with their PBL problem. A lecture can also be a part of student-directed learning if students request for it because they decided it is critical to their learning at that time [2]. An interactive approach to stimulate thinking can be introduced by pausing the lecture at key points to direct questions at pairs (or groups) of students to foster active processing of their pre-understanding [42,43]. Lectures are compatible with standard PBL if they complement the trigger learning objectives or are delivered in congruence with the tenets of PBL and do not displace time for self directed learning.

### 3. Preparation

PBL was implemented at Maastricht and Newcastle only after some of their staff spent months to years learning it at McMaster [6]. Walton and Matthews have cautioned that the crafting of a PBL curriculum demands a major planning effort because the multidisciplinary nature of PBL problems requires collaboration across departments [17]. Preparation for McMaster’s pioneering curriculum took 4 years [6] while Sherbrooke’s conversion to PBL needed 3 years of preparation [44]. Both Sherbrooke [9] and the University of Hong Kong [45] reported the crucial role of a core group of staff who pushed for curriculum reform. They coordinated staff training in PBL conducted by internal and external experts. The importance of such training is underscored in Hong Kong where 268 tutors and 57 case-writers were trained over a 3-year period, and priority placed on continued refresher PBL workshops [46]. Specialised training of this nature is best coordinated by a dedicated staff development unit whose representative should be present at management meetings to ensure decisions affecting the school are favourable to PBL [47]. A key factor for Sherbrooke’s successful conversion was staff acceptance of student-centered pedagogy, achieved by having a majority of them attend training programs in medical education and PBL which were initiated long before the curricular change [9]. Likewise at Liverpool, the staff development program for PBL started 2 years before PBL was implemented [10]. Staff who are planning for curricular conversion to standard PBL need to set a timeframe in the order of years to allow sufficient time for designing the curriculum, constructing the PBL problems and educating the staff in medical pedagogy and PBL skills.

### 4. Leadership

The effective establishment and maintenance of a PBL curriculum requires a raft of supporting policies such as funding staff visits to prominent PBL centres, recognition and reward for staff involvement in PBL, provision of equipped PBL rooms and a student assessment system compatible with PBL. Hence conversion to standard PBL requires the endorsement, and at times the enforcement of a PBL advocate who holds administrative authority in the school and/or the university. This is particularly necessary when staff are required to make difficult changes such as reduction or removal of lectures. The staff in a hybrid PBL school are often divided by curricular philosophy such that the balance of opinion may change with the departure of PBL enthusiasts or arrival of traditionalists. Des Marchais has warned that “Educational reform demands strong and innovative leadership, because the pressure to revert to tradition is always present and may erode the system even as it is put in place” [44]. Hence strong supportive leadership is required for successful PBL curricular change [9,46,48].

Another important leadership role for supporting the conversion of hybrid curricula to standard PBL is that which can be played by PBL organizations. Schools intending to adopt PBL but lacking curriculum development expertise will need help in practicalities such as the frequency of tutorial sessions, the number of students per tutorial group and the hours of self directed learning per PBL problem. There are no strict rules governing these issues but information on regional best practices will be a helpful starting point. They would also want to know the established PBL schools that are prepared to host staff from other schools who wants to learn from them. A regional medical education or PBL association would be well placed as the central channel for such information.

## Summary

PBL has revolutionized medical education because it fosters the skills required by practising physicians. Neville’s review of the 40 years of PBL implementation predicts the PBL curriculum of the future to be a hybrid in which “students are prepared didactically with fundamental concepts on which they elaborate in small-group tutorials” [49]. This is standard PBL, where all curricular inputs support the learning driven by problems. Consequently the oft-used term ‘hybrid PBL’ should refer to all other curricular configurations involving PBL tutorials not amounting to standard PBL. Hybrid PBL curricula without oversight by medical education experts can degenerate into dysfunctional curricula inferior even to the traditional approach from which PBL emerged. Such schools should inspect their curriculum periodically for signs of dysfunction to enable timely corrective action. A decision to convert fully to standard PBL can be cost feasible, but will require time, expertise and commitment which is only sustainable with supportive leadership.

## Ethical approval

Not applicable.

## Abbreviation

TBL: Team-based learning.

## Competing interests

The authors declare that they have no competing interests.

## Author’s contributions

WL conceived and drafted the manuscript.

## Pre-publication history

The pre-publication history for this paper can be accessed here:

http://www.biomedcentral.com/1472-6920/12/89/prepub

## References

[B1] Maudsley G Do we all mean the same thing by ‘problem-based Learning’? a review of the concepts and a formulation of the ground rules Acad Med 1999 74 178 185 10065058 10.1097/00001888-199902000-00016

[B2] Barrows H Tamblyn R Problem-based learning: an approach to medical education 1980 New York: Springer

[B3] Barrett T Barrett T, Labhrainn IM, Fallon H Understanding problem-based learning Handbook of enquiry and problem-based learning 2005 Galway: CELT 13 25

[B4] Savery JR Duffy TM Wilson BG Problem based learning: an instructional model and its constructivist framework Constructivist learning environments: case studies in instructional design 1996 New Jersey: Educational Technology Publications 135 150

[B5] Koh GC-H Khoo HE Wong ML Koh D The effects of problem-based learning during medical school on physician competency: a systematic review CMAJ 2008 178 34 41 18166729 10.1503/cmaj.070565 PMC2151117

[B6] Barrows HS Problem-based learning applied to medical education 2000 Springfield: Southern Illinois University School of Medicine Press

[B7] Donner RS Bickley HB Problem-based learning in American medical education: an overview Bull Med Libr Assoc 1993 81 294 298 8374585 PMC225793

[B8] Armstrong EG Boud D, Feletti GE A hybrid model of problem-based learning The challenge of problem-based learning 1997 2 London: Kogan Page 137 150

[B9] Grand’Maison P Marchais JED Preparing faculty to teach in a problem-based learning curriculum: the sherbrooke experience CMAJ 1991 144 557 562 1998902 PMC1452841

[B10] Bligh J Wilkinson P Report of a workshop on problem-based learning and its implications for medical education in the UK Postgrad Med J 1997 73 449 459 9338044 10.1136/pgmj.73.861.449 PMC2431402

[B11] Kinkade S A snapshot of the status of problem-based learning in U.S. Medical schools, 2003–04 Acad Med 2005 80 300 301 15734817 10.1097/00001888-200503000-00021

[B12] Oda Y Koizumi S Status of medical education reform at saga medical school 5 years after introducing PBL Kaohsiung J Med Sci 2008 24 S46 S53 18364287 10.1016/S1607-551X(08)70094-9 PMC11922112

[B13] Tsou K-I Short-term outcomes of a near-full PBL curriculum in a new Taiwan medical school Kaohsiung J Med Sci 2009 25 282 293 19502151 10.1016/S1607-551X(09)70075-0 PMC11917576

[B14] Blumberg P Michael JA Development of self-directed learning behaviors in a partially teacher-directed problem-based learning curriculum Teach Learn Med 1992 4 3 8

[B15] Houlden RL Collier CP Frid PJ John SL Pross H Problems identified by tutors in a hybrid problem-based learning curriculum Acad Med 2001 76 81 11154202 10.1097/00001888-200101000-00021

[B16] Taylor D Miflin B Problem-based Learning: where are we now? Med Teach 1998 30 742 763 18946818 10.1080/01421590802217199

[B17] Walton HJ Matthews MB Essentials of problem-based learning Med Educ 1989 23 542 558 2593886 10.1111/j.1365-2923.1989.tb01581.x

[B18] Albanese MA Mitchell S Problem-based learning: a review of literature on its outcomes and implementation issues Acad Med 1993 68 52 81 8447896 10.1097/00001888-199301000-00012

[B19] Kwan C-Y Tam L Commentary: hybrid PBL- what is in a name? J Med Educ 2009 13 157 165

[B20] Sefton AJ Kwan C-Y Problem-based learning: Impact and implementation in a workshop setting J Med Educ 2002 6 134 142

[B21] Wood DF ABC of learning and teaching in medicine: problem-based learning BMJ 2003 326 328 330 12574050 10.1136/bmj.326.7384.328 PMC1125189

[B22] Weiss RE Designing problems to promote higher-order thinking New Dir Teach Learn 2003 95 25 31

[B23] Lechky O U of T not the only Ontario medical school heavily involved in curriculum renewal CMAJ 1992 147 1233 1237 1393940 PMC1336501

[B24] Liaison Committee on Medical Education Functions and structure of a medical school. Standards for accreditation of medical education programs leading to the M.D. Degree 2012 Washington: Liaison Committee on Medical Education

[B25] General Medical Council Tomorrow’s Doctors 2009 London: General Medical Council

[B26] Tärnvik A Revival of the case method: a way to retain student-centred learning in a post-PBL era Med Teach 2007 29 e32 e36 17538830 10.1080/01421590601039968

[B27] Pearson TA Barker WH Fisher SG Trafton SH Integration of the case-based series in population-oriented prevention into a problem-based medical curriculum Am J Prev Med 2003 24 4S 10.1016/s0749-3797(03)00030-8 12744987

[B28] Srinivasan M Wilkes M Stevenson F Nguyen T Slavin S Comparing problem-based learning with case-based learning: effects of a major curricular shift at Two institutions Acad Med 2007 82 74 82 17198294 10.1097/01.ACM.0000249963.93776.aa

[B29] Parmelee D Michaelsen LK Cook S Hudes PD Team-based learning: a practical guide: AMEE guide No. 65 Med Teach 2012 34 e275 e287 22471941 10.3109/0142159X.2012.651179

[B30] Okubo Y Team-based learning, a learning strategy for clinical reasoning, in students with problem-based learning tutorial experiences Tohoku J Exp Med 2012 227 23 29 22516766 10.1620/tjem.227.23

[B31] Abdelkhalek N Hussein A Gibbs T Hamdy H Using team-based learning to prepare medical students for future problem-based learning Med Teach 2010 32 123 129 20163227 10.3109/01421590903548539

[B32] Walsh K Walsh K Cost effectiveness in medical education: an introduction Cost effectiveness in medical education 2010 London: Radcliffe 1 4

[B33] Finucane P Shannon W McGrath D The financial costs of delivering problem-based learning in a new, graduate-entry medical programme Med Educ 2009 43 594 598 19493185 10.1111/j.1365-2923.2009.03373.x

[B34] Sefton AJ From a traditional to a problem-based curriculum- estimating staff time and resources Educ Health 1997 10 165 178

[B35] Martinez W Azzam A Mack K Student near-peer co-tutors in PBL groups Med Educ 2009 43 475 476 19422498 10.1111/j.1365-2923.2009.03315.x

[B36] Margetson D Boud D, Feletti GE Why is problem-based learning a challenge? The challenge of problem-based learning 1997 2 London: Kogan Page 36 44

[B37] Mcmanus C New pathways to medical education: learning to learn at Harvard medical school BMJ 1995 311 67

[B38] Karlsen KA Vik T Westin S The problem-based medical curriculum in Trondheim- did it turn out as planned? Tidsskr Nor Laegeforen 2000 120 2269 2273 10997086

[B39] Hamdy H The fuzzy world of problem-based learning Med Teach 2008 30 739 741 18946817 10.1080/01421590802345891

[B40] Azer SA What makes a great lecture? Use of lectures in a hybrid PBL curriculum Kaohsiung J Med Sci 2009 25 109 115 19419915 10.1016/S1607-551X(09)70049-X PMC11917756

[B41] van Berkel H Schmidt H On the additional value of lectures in a problem-based curriculum Educ Health 2005 18 45 61 10.1080/13576280500042648 15804645

[B42] Fyrenius A Bergdahl B Silen C Lectures in problem-based learning- Why, when and how? an example of interactive lecturing that stimulates meaningful learning Med Teach 2005 27 61 65 16147772 10.1080/01421590400016365

[B43] Nayak SB The broken lecture: an innovative method of teaching Advan Physiol Educ 2006 30 48 16481611 10.1152/advan.00047.2005

[B44] Des Marchais JE A student-centered, problem-based curriculum: 5 years’ experience CMAJ 1993 148 1567 1572 8477383 PMC1491821

[B45] MacKinnon MM Conversion to problem-based learning: a comparison of three approaches to curriculum reform Cornerstones: proceedings of the 1999 annual HERDSA conference: 12–15 July 1999; Melbourne 1999 Melbourne: University of Melbourne

[B46] Tang G Quality assurance of problem-based learning (PBL): the Hong Kong experience Ann Acad Med Singapore 2001 30 363 365 11503540

[B47] Kolmos A Premises for changing to PBL Int J Scholarship Teach Learn 2010 4 1 http://academics.georgiasouthern.edu/ijsotl/v4n1/invited_essays/PDFs/Invited_Essay_Kolmos.pdf.

[B48] Achike FI Sustaining the effectiveness of PBL in a medical curriculum J Med Educ 2003 7 92 96

[B49] Neville AJ Problem-based learning and medical education forty years on Med Princ Pract 2009 18 1 9 19060483 10.1159/000163038

